# Genetic variants in IL1A and IL1B contribute to the susceptibility to 2009 pandemic H1N1 influenza A virus

**DOI:** 10.1186/1471-2172-14-37

**Published:** 2013-08-08

**Authors:** Yingxia Liu, Shaoyuan Li, Guoliang Zhang, Guang Nie, Zhizhong Meng, Dongting Mao, Chang Chen, Xinchun Chen, Boping Zhou, Gucheng Zeng

**Affiliations:** 1 Guangdong Key Laboratory for Emerging Infectious Diseases, Shenzhen Third People’s Hospital, Guangdong Medical College, Shenzhen, China; 2 Department of Microbiology, Zhongshan School of Medicine, Key Laboratory for Tropical Diseases Control of the Ministry of Education, Sun Yat-sen University, Guangzhou, China; 3 School of Bioscience and Bioengineering, South China University of Technology, Guangzhou, China

**Keywords:** Interleukin-1, H1N1, Influenza, Single-nucleotide polymorphisms (SNPs)

## Abstract

**Background:**

Host genetic variations may contribute to disease susceptibility of influenza. IL-1A and IL-1B are important inflammatory cytokines that mediate the inflammation and initiate the immune response against virus infection. In this study, we investigated the relationship between single-nucleotide polymorphisms (SNPs) of Interleukin-1A (IL-1A) and Interleukin-1B (IL-1B) and the susceptibility to 2009 pandemic A/H1N1 influenza (A(H1N1)pdm09). 167 patients whom were confirmed with A(H1N1)pdm09 and 192 healthy controls were included in this study. Four SNPs (rs1304037, rs16347, rs17561, rs2071373) in IL1A gene and three SNPs (rs1143623, rs3917345, rs1143627) in IL1B gene were genotyped by using matrix-assisted laser desorption/ionization time-of-flight (MALDI-TOF) mass spectrometry platform, and the associations of the genetic variants of IL-1 with susceptibility to A(H1N1)pdm09 were then assessed.

**Results:**

The polymorphisms of rs17561 in IL1A gene and rs1143627 in IL1B gene were found to be associated with susceptibility to A(H1N1)pdm09 with P values of 0.003 (OR 2.08, 95% CI 1.27-3.41) and 0.002 (OR 1.62 , 95% CI 1.20-2.18), respectively. However, no significant difference in allelic frequency was observed for other SNPs between cases and controls.

**Conclusions:**

This study provides a new insight into pathogenesis of A(H1N1)pdm09, suggesting that genetic variants of IL-1A and IL-1B may exert a substantial impact on the susceptibility of A(H1N1)pdm09 virus infection.

## Background

The global pandemic caused by the novel A(H1N1)pdm09 was first declared in Mexico and the United States in April 2009, inducing severe morbidity and mortality in a subset of the population [1]. This novel strain of influenza A virus is made up of a unique combination of gene segments from Eurasian swine, human and avian influenza viruses [2]. Thus, seasonal influenza vaccines do not confer effective cross-protection against A(H1N1)pdm09 virus infection. While the virulence and evolution of influenza virus have been intensively studied, the impact of host genetic background on the susceptibility to pandemic influenza virus infection remains largely unknown.

It has been suggested that the host genetic factors may affect the susceptibility and progression of microbial infections [3]. For example, Chinese population-attributable risk for severe influenza infection was ten-fold greater than Northern European population-attributable risk because of the high frequency of rs12252-C in Han Chinese [4]. Meanwhile, the SNPs in TLR3 [5], CD55 [6], C1q and FCGR2A [7], TNF [8,9], LTA, IL8 and IL1B [9] were suggested to be connected with the severity of A(H1N1)pdm09 virus infection.

During influenza virus infection, IL-1B has been demonstrated to mediate acute pulmonary inflammatory pathology [10]. Pro-IL-1B was cleaved by caspase-1 which was activated through the formation of the NLRP3 inflammasome [11]. Recent studies have shown that IL-1A secretion was also regulated by the NLRP3 inflammasome [12,13]. In addition, IL-1A and IL-1B induced the expression of a variety of inflammatory mediators, which may initiate the cascade of inflammatory responses and induced the activation of T cells [14,15]. To date, few studies have explored the association between IL-1 polymorphisms and A/H1N1 susceptibility [9]. Therefore, investigation of whether the genetic variants in IL-1 impact the susceptibility to A(H1N1)pdm09 is of great importance.

In this study, we examined 7 SNPs in IL1 gene and evaluated their association with A(H1N1)pdm09 susceptibility. The results indicated that rs17561 polymorphism in IL1A gene and rs1143627 polymorphism in IL1B gene, two novel SNPs of IL-1 gene, contributed to the susceptibility to A(H1N1)pdm09.

## Methods

### Study subjects and samples

167 patients with A(H1N1)pdm09 were recruited in Shenzhen Third People’s Hospital, China during 2009 influenza pandemic period. 167 patients (91 males and 76 females) were included in this study, and the average age of patients is 22.37 (SD = 11.95). The diagnosis of A(H1N1)pdm09 was followed the guideline released by the Ministry of Health of the People’s Republic of China. 192 Healthy donors (HD) were from Shenzhen, China during the same period, and no occurrence of A(H1N1)pdm09 was found in the HD during 1 year’s follow-up. 192 health individuals (101 males and 91 females) were recruited as controls, and the average age of healthy individuals is 24.53 (SD = 10.29). All subjects were Chinese Han people whom have received flu vaccine previously. Venous blood was drawn from all subjects after an overnight fasting. Blood corpuscles were stored at −80°C for DNA purification. The study was approved by the Institutional Review Board of Shenzhen Third People’s Hospital, and informed consent was obtained from each participant. There is no significant difference for the average age and gender distribution between cases and controls (P >0.05).

### DNA extraction and SNP selection

Genomic DNA was obtained from peripheral blood samples, using the QIAamp DNA Blood Mini kit (Qiagen, Hilden, Germany) according to the manufacturer’s instruction. Isolated DNA was stored at −80°C before usage. SNPs were selected according to methods described previously [16], with focusing on their potential regulatory roles, such as transcription binding sites in the promoter region, microRNA target sites in the 3′ untranslated region (UTR), protein phosphorylation sites in the extrons and other putative regulation sites.

To search for the SNPs with alleles that alter the putative transcription factor binding sites, we scanned the promoter region of the gene in TRANSFAC database [17]. For the annotated SNPs in dbSNP129 that are within 2000 bp upstream to 500 bp downstream of the IL-1 gene, we extracted their flanking sequence (1-25 bp) from the dbSNP website (http://www.ncbi.nlm.nih.gov/projects/SNP/). We then used a PWM_SCAN Algorithm [18] to scan each sequence in the set to test whether it had a putative binding site (PBS), using the method described previously [19]. To search for the SNPs with alleles that alter the putative microRNA target sites, we first searched the PITA database for the microRNAs that could target the 3′ UTR region of the IL-1 gene. For the SNP that is in the target site, we obtained its flanking sequence (1-50 bp) from the UCSC genome browser according to its genomic location. We then tested whether the change of alleles also changed the microRNA target interaction by RNA hybridization. In addition, we used tagging procedure to select SNPs that cover the IL-1 gene [20], and chose the CHB + JPT HapMap panel, r^2^ = 0.8 and default settings for all other parameters. We also selected several other SNPs in the IL-1 gene region that were reported to be associated with disease severity [21-23]. The allelic variant substitution bases and the allele specific primer sequences were shown in Table 1.

**Table 1:** Outline of primers for IL1A, IL1B SNP genotyping

**Gene**	**SNP ID**	**Polymorphisms**	**Forward primer**	**Reverse primer**	**Un-extension primer**
			**(5′-3′)**	**(5′-3′)**	**(5′-3′)**
IL1A	rs1304037	A > G	ACGTTGGATGTCGATTAAGAGTTCATCAGC	ACGTTGGATGAGCCACAGACCTAGGATTTC	AGTTCATCAGCAACTTAAAAG
	rs16347	DEL > TGAA	ACGTTGGATGGTAGGACTTGATTGCAGGTG	ACGTTGGATGTTGAGCCAGTAATTGGTCCG	TGCAGGTGGAATTGAA
	rs17561	G > T	ACGTTGGATGGGTTTTAGAAATCATCAAGCC	ACGTTGGATGGAATTCGTATTTGATGATCC	TCATCAAGCCTAGGTCA
	rs2071373	C > T	ACGTTGGATGTGATGTGCATTGGCTTCTCC	ACGTTGGATGAACATCCTGATGAAGCCTGC	TCCCAGAACAGAGCAGAAC
IL1B	rs1143623	G > C	ACGTTGGATGATGTGCCAGGTATCGTGCTC	ACGTTGGATGACCTATTTCCCTCGTGTCTC	GCTCGCTCTGCATTAT
	rs3917345	DEL > TGGT	ACGTTGGATGTGCTGGTGTCTCGGTTAAAG	ACGTTGGATGTCTGAGACTCTATCTCTTGG	AGAGAAACTGATAACTCTTGGT
	rs1143627	T > C	ACGTTGGARGCCTCGAAGAGGTTTGGTATC	ACGTTGGATGTCTAGCCTCCTACTTCTGC	ATCCCTCGCTGTTTTTAT

### Genotyping

SNPs’ genotyping was based on analysis of primer extension products generated from previously amplified genomic DNA using a chip-based MALDI-TOF mass spectrometry platform (Sequenom, San Diego, CA). The amplification PCR primer pairs and the extend primers were designed by MassARRAY assay designer software v3.1, in combination with manual adjustment (Table 1). PCR amplification was performed in the HotStar buffer system (Qiagen, Hilden, Germany). Extend products were dispensed onto a 384-spot plate, and Thermo cycling (Applied Biosystems, USA) was initiated at 94°C for 5 min; followed by 40 cycles of 94°C for 30 s, 55°C for 30 s, 72°C for 30 s; and a final extension of 72°C for 3 min to amplify identical length products. Following genomic amplification of the target regions, excess deoxyribonucleotide triphosphates were dephosphorylated with 2 μL shrimp alkaline phosphatase by incubation at 37°C for 60 min, and stopped by inactivation at 85°C for 10 min.

The selected SNPs were analyzed in one primer-extension reaction, the PCR was then performed at 94°C for 15 min, 40 cycles of 94°C for 5 s, (52°C for 5 s, and 80°C for 5 s, 5 cycles), followed by a final extension done at 72°C for 3 min. After desalting extension products for MALDI-TOF mass spectrometry analysis by the addition of 16 μL water and 6 mg of clean resin to each sample. Data analysis was performed by using MassARRAY typer software 4.0.

### Statistical analysis

The frequencies of allele and genotype were determined by direct counting. All SNPs investigated in this study were tested for Hardy-Weinberg equilibrium. The Pearson *χ*2 test was used to compare allele and genotype distribution in cases and controls. Odds ratios (ORs) and their 95% confidence intervals (CI) were calculated with Miettinen’s method. Each SNP was calculated under four alternative models (dominant, recessive, multiplicative and additive), as previously described [4,24-26]. Bonferroni correction was used to compare the allelic frequency between participants with different genotypes. All the statistical procedures were performed with SPSS13.0 software. P <0.05 was considered to be significant.

## Results

### Selection and genotyping of IL1A/IL1B SNPs

**Figure 1:**
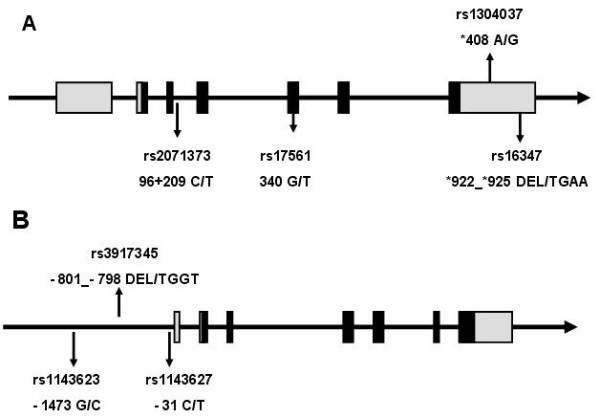
**Schematic representation of IL-1α and IL-1β genes. (A)** is IL-1α and **(B)** is IL-1β gene. The boxes represent the exons. The black boxes represent coding sequences and grey boxes represent 3′ or 5′ untranslated regions. SNPs analyzed in this study are marked with an arrow. The first line following the direction of arrow marking the SNPs shows the SNP name (i.e. rs-number) from NCBI, and the second line shows the SNP position and the base substitution starting from the start codon.

**Figure 2:**
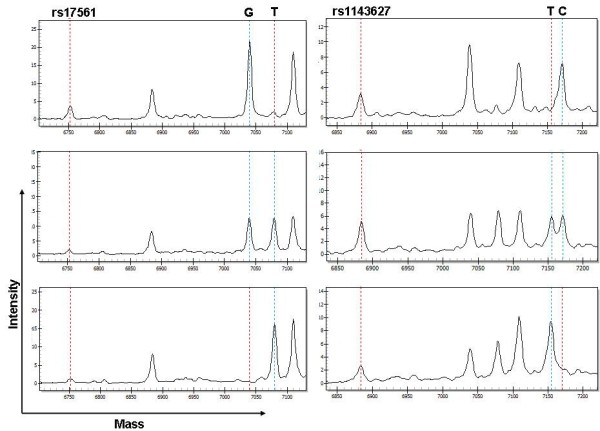
**MALDI–TOF mass spectrum–based analysis of rs17561 and rs1143627 SNPs.** Typical MALDI-TOF MS spectra from three DNA samples are shown. Dashed lines mark the unextended primers and product/extended primers. Rs17561 SNP includes GG, GT, and TT genotypes, and rs1143627 SNP includes CC, TC, and TT genotypes.

To choose relevant SNPs for IL1A and IL1B gene, we considered about 100 known and rare variants, which are publicly available in database (dbSNP: http://www.ncbi.nlm.nih.gov/SNP/; CYPallele nomenclature: http://www.cypalleles.ki.se). After bioinformatic analysis and prediction for SNPs that may influence gene expression or protein structure and function, a total of 7 SNPs, including 4 in the IL1A gene (rs17561 G > T, rs1304037 A > G, rs2071373 C > T, rs16347 DEL > TGAA), and 3 in the IL1B gene (rs1143623 G > C, rs3917345 DEL > TGGT, rs1143627 T > C), were selected finally. The locations of these SNPs in IL-1 were shown in Figure 1. Rs2071373 is in the intron 3 and 209 bp after the extron 3 (the length from the coding sequence start site to the extron 3 is 96 bp) of IL1A gene. Rs17561 is in the extron 5 of IL1A gene and 340 bp after coding sequence (CDS) start site. Rs1304037 is in the 3′UTR of IL1A gene and 408 bp after CDS terminal site. Rs16347 is in the 3′UTR of IL1A gene, 922 bp after CDS terminal site and a 4 bp fraction is missing. 3 SNPs of IL1B gene are all in the promoter region. Rs1143623 is 1473 bp before the transcription start site of IL1B gene. Rs3917345 is 798 bp before the transcription start site of IL1B gene and a 4 bp fraction is missing. Rs1143627 is 31 bp before the transcription start site of IL1B gene. Genotypes of these 7 SNPs were determined using the MALDI-TOF mass spectrometry platform in a cohort of 167 cases and 192 controls. The intensities of mass signal of rs17561 and rs1143627 SNPs from three DNA samples analyzed by MALDI-TOF mass spectrum were shown in Figure 2.

### Association of IL1A gene SNPs with A/H1N1 susceptibility

The genotypic frequencies of the two groups in these 4 SNPs were shown in Table 2. The distribution of genotype in patients with pH1N1/09 and healthy controls were coincident with Hardy-Weinberg equilibrium (P > 0.05). The T allele of rs17561 showed a two-fold increase in risk for influenza compared with the G allele (multiplicative model, P = 0.003, odds ratio, OR = 2.08, 95% confidence interval, CI 1.27-3.41). Under the dominant model, heterozygotes and homozygotes for the T allele had a two-fold increase of risk for influenza compared with homozygotes for the G allele (dominant model, P = 0.001, OR = 2.43, 95% CI 1.42-4.15). However, no significant differences in allelic frequencies for other SNPs were found between cases and controls. Thus, these data suggested that the rs17561 SNP, located on the extron of IL1A leading to Ser114Ala mutation, was associated with A(H1N1)pdm09 susceptibility.

**Table 2:** Distribution of genotype and allele frequencies of IL1A SNP

**Gene**						**Different genetics models**					
**IL1A**		**Controls**	**Cases**	**Multiplicative**		**Additive**		**Dominant**		**Recessive**	
**SNP ID**	**Genotype**	**No (%)**	**No (%)**	**P value**	**OR**	**P value**	**OR (95% CI)**	**P value**	**OR (95% CI)**	**P value**	**OR (95% CI)**
RS1304037	AA	161 (83.9)	130 (77.8)	0.262	1.32 (0.81-2.15)			0.147	1.48 (0.87-2.51)	0.386	0.38 (0.04-3.69)
	AG	28 (14.6)	36 (21.6)			0.223	0.26 (0.03-2.63)				
	GG	3 (1.5)	1 (0.6)			0.432	0.41 (0.04-4.02)				
rs16347	DEL/DEL	81 (42.2)	68 (40.7)	0.482	1.12 (0.82-1.51)			0.778	1.06 (0.70-1.62)	0.323	1.36 (0.74-2.48)
	DEL/TGAA	88 (45.8)	73 (43.7)			0.343	1.36 (0.72-2.59)				
	TGAA/TGAA	23 (12.0)	26 (15.6)			0.367	1.35 (0.70-2.57)				
rs17561	GG	166 (86.5)	121 (72.5)	**0.003**	2.08 (1.27-3.41)			**0.001**	2.43 (1.42-4.15)	0.646	0.54 (0.05-6.37)
	GT	24 (12.5)	45 (26.9)			0.260	0.27 (0.02-3.10)				
	TT	2 (1.0)	1 (0.6)			0.758	0.69 (0.06-7.66)				
rs2071373	CC	80 (41.7)	68 (40.7)	0.443	1.13 (0.83-1.53)			0.856	1.04 (0.68-1.59)	0.194	1.51 (0.81-2.84)
	CT	92 (47.9)	74 (44.3)			0.191	1.55 (0.80-3.02)				
	TT	20 (10.4)	25 (15.0)			0.259	1.47 (0.75-2.88)				

*CI*, confidence interval; *OR*, odds ratio. The P values <0.05 were highlighted in boldface.

### Association of IL1B gene SNPs with A/H1N1 susceptibility

3 SNPs in IL1B were successfully genotyped and considered for genetic association tests (Table 3), and all these SNPs were consist with Hardy-Weinberg equilibrium (P >0.05). The rs1143627 SNP located on 31 base pairs upstream from the transcription start site of IL1B gene was evaluated for the association with increased risk of influenza susceptibility. Under the multiplicative model, the T allele showed an increase in risk for influenza compared with the C allele (multiplicative model, P = 0.002, OR = 1.62, 95% CI 1.20-2.18). Under the additive model, genotype TT had a two-fold higher risk for influenza compared with genotype CC (additive model, P = 0.005, OR = 2.38, 95% CI 1. 92–4.40). Under the dominant model, heterozygotes and homozygotes for the T allele had a two-fold higher risk for influenza compared with homozygotes for the C allele (dominant model, P = 0.002, OR = 1.99, 95% CI 1.28-3.09). Other 2 SNPs in IL1B showed no significant differences in allelic frequency among cases and controls. Thus, rs1143627 SNP in IL1B gene showed significant association with increased susceptibility to influenza.

**Table 3:** Distribution of genotype and allele frequencies of IL1B SNP

**Gene**						**4 statistical models**					
**IL1B**		**Controls**	**Cases**	**Multiplicative**		**Additive**		**Dominant**		**Recessive**	
**SNP ID**	**Genotype**	**No (%)**	**No (%)**	**P value**	**OR (95% CI)**	**P value**	**OR (95% CI)**	**P value**	**OR (95% CI)**	**P value**	**OR (95% CI)**
rs1143623	GG	57 (29.7)	37 (22.2)	0.106	1.27 (0.95-1.71)			0.105	1.48 (0.92-2.40)	0.318	1.28 (0.79-2.07)
	GC	92 (47.9)	85 (50.9)			0.633	1.13 (0.68-1.89)				
	CC	43 (22.4)	45 (26.9)			0.11	1.61 (0.90-2.90)				
rs3917345	DEL/DEL	191 (99.5)	166 (99.4)	0.541	1.15 (0.07-18.47)			0.541	1.15 (0.07-18.55)	—	—
	DEL/TGGT	1 (0.5)	1 (0.6)			—	—				
	TGGT/TGGT	0 (0.0)	0 (0.0)			—	—				
rs1143627	TT	84 (43.8)	47 (28.1)	**0.002**	1.62 (1.20-2.18)			**0.002**	1.99 (1.28-3.09)	0.063	1.68 (0.97-2.91)
	TC	81 (42.2)	84 (50.3)			0.399	1.29 (0.72-2.31)				
	CC	27(14.1)	36 (21.6)			**0.005**	2.38 (1.29-4.40)				

*CI*, confidence interval; *OR*, odds ratio. The P values <0.05 were emphasized in boldface.

## Discussion

In this study, we analyzed the genetic factors impacting the susceptibility to A(H1N1)pdm09. We examined 7 SNPs in IL1A and IL1B genes and evaluated the association between SNPs of IL-1 and A(H1N1)pdm09 susceptibility. We found that rs17561 polymorphism in IL1A gene and rs1143627 polymorphism in IL1B gene contributed to the susceptibility to A(H1N1)pdm09.

In humans, IL-1 exists in two forms, IL-1A (encoded by IL1A gene) and IL-1B (encoded by IL1B gene), and both forms of IL-1 locate on chromosome 2 [27]. IL-1A and IL-1B are inflammatory cytokines that play important roles in recruitment of the immune and inflammatory cells and development of adaptive immune responses [28]. Accumulating evidence has suggested that IL-1A and IL-1B play important roles in innate immunity against viral infection. Infection of viruses such as, hepatitis A virus (HAV) [29], epstein-barr virus (EBV) [30], HIV [31] and human papilloma viruses (hrHPVs) [3], may activate the extracellular signal and induce production of IL-1. Several studies have documented an early rise in IL-1 in bronchoalveolar lavage (BAL) fluids or lung homogenates in temporal association with symptom formation and lung pathology after infection with A/PR/8/34 H1N1 or A(H1N1)pdm09 [32-34].

In fact, IL1A with rs17561 allele T has been suggested to be associated with immunopathogenesis of several diseases, including nasal polyposis [22], chronic rhinosinusitis [23]. Rs17561 polymorphism was related with high levels of C-reactive protein, which can regulate the severity inflammatory response [35]. Interestingly, our result also indicated that rs17561 allele T increased the risk of A(H1N1)pdm09 susceptibility. In contrast, rs1304037 and rs16347 locating on 3′UTR, and rs2071373 locating on intron box did not display any association with the susceptibility to A(H1N1)pdm09. The SNP rs17561 represents a nonsynonymous mutation (Ala114Ser) in IL-1A protein, suggesting that this mutation may lead to a potential functional variation in host susceptibility to A(H1N1)pdm09. However, the exact mechanism needs to be further studied.

During influenza virus infection, the immune response was triggered by the influenza virus ion channel M2 which was essential for virus entry and replication, leading to the assembly of inflammasome in macrophages and dendritic cells (DCs) [36]. Then, the activation of inflammasome resulted in the cleavage of pro-IL-1B by caspase-1 and produced the mature form of IL-1B [37]. IL-1B may act with IL-6 to induce IFN-γ production by T cells [14] and promote RORγt expression and Th-17 polarization of CD4 T cells [15]. The Th-17 effector cells produced IL-17 and facilitated the recruitment of neutrophils and inflammation [38].

Here, we found a significantly high frequency of IL-1B rs1143627 allele C in A/H1N1 patients, suggesting the hosts with allele C were more susceptible to this virus infection. Importantly,the frequency of the rs1143627 allele C is quite rich in Han Chinese (minor allele frequency = 0.476). Thus, elucidation of the underlying mechanism of the polymorphism of rs1143627 is of great importance for in-depth understanding of the susceptibility of influenza in Chinese population. Rs1143627 polymorphism involved the variation of IL-1B −31 allele C to T and is located on the TATA box of IL1B gene promoter region, which has been demonstrated to be a critical motif partially determining IL-1B transcriptional efficiency [39]. Furthermore, the rs1143627 and its franking sequences can be bound by transcription factors, including c/EBPβ, TBP and Spi-1 [40,41]. Electrophoretic mobility shift assay (EMSA) analysis suggested that c/EBPβ confers a much higher affinity to rs1143627-C probe than to T probe [40]. Several reports have shown that the allele T of rs1143627 enhanced IL-1B protein expression [40,42], and T-allele of rs1143627 was considered to be a pro-inflammatory allele [41]. People who carry allele T had a higher IL-1B expression that may promote the production of IFN-γ, which plays an important role in virus clearance [14]. IL-1B may also facilitate the differentiation and proliferation of pro-inflammatory cells such as Th-17 in humans [15,43]. In contrast, the expression of IL-1B may be much lower in people who carry allele C, leading to weaker immune response during viral infection.

Thus, it is possible that IL1B SNP rs1143627 C allele has a lower binding affinity to c/EBPβ and reduced levels of IL-1B expression, which may therefore weaken the inflammatory response of Th-17 and Th-1 cells in humans during influenza infection. A cohort of study with a larger population size is needed to further confirm the association of IL1A rs17561 allele T and IL1B rs1143627 allele C with an increased risk for the susceptibility to A(H1N1)pdm09. Functional studies will also be needed to further investigate the role and mechanism of genetic variants of IL-1 on the impact of susceptibility to A(H1N1)pdm09.

## Conclusion

IL1A rs17561 allele T and IL1B rs1143627 allele C may confer an increased risk for the susceptibility to A(H1N1)pdm09. Thus, this study provides a new insight into the immunopathogenesis of influenza A virus infection in humans.

## Abbreviations

SNPs: Single-nucleotide polymorphisms; IL: Interleukin; A(H1N1)pdm09: 2009 pandemic A/H1N1 influenza; MALDI-TOF: Matrix-assisted laser desorption/ionization time-of-flight; OR: Odds ratio; CI: Confidence interval.

## Competing interests

The authors declare that they have no conflict of interests.

## Authors’ contributions

YL, SL GZ and GN collected the samples, performed the experiments and analyzed the data. ZM, DM and CC analyzed the data. XC, BZ and GZ oversaw the research and wrote the manuscript. All authors read and approved the final manuscript.

## Authors’ information

Y Liu, MD, PhD, Director, S Li, BS, G Zhang, MD, PhD, G Nie, PhD, Z Meng, PhD, D Mao, BS, C Chen, BS, X Chen, MD, PhD, B Zhou, MD, PhD, Dean, G. Zeng, PhD.
